# Combinations of lifestyle behaviors and cancer risk among Korean adults

**DOI:** 10.1038/s41598-023-40819-w

**Published:** 2023-08-23

**Authors:** Ngoc Minh Luu, Thi Tra Bui, Thi Phuong Thao Tran, Thi Huyen Trang Nguyen, Jin-Kyoung Oh

**Affiliations:** 1 Department of Cancer Control and Population Health, National Cancer Center Graduate School of Cancer Science and Policy, 323 Ilsan-ro, Ilsandong-gu, Goyang-si, Gyeonggi-do 410-769 Republic of Korea; 2 Department of Research Methodology and Biostatistics, School of Preventive Medicine and Public Health, Hanoi Medical University, Hanoi, Vietnam; 3 Division of Cancer Prevention, National Cancer Control Institute, National Cancer Center, Goyang, Republic of Korea

**Keywords:** Cancer prevention, Risk factors, Epidemiology

## Abstract

Combinations of lifestyle behaviors may lead to different cancer risks. This study aimed to identify the latent classes based on lifestyle behavior trajectories and to investigate the association between these latent classes and cancer risk. Participants in the 2002–2003 National Health Insurance Service general health examination were included. Data on smoking, alcohol drinking, body mass index (BMI), and physical activity measured four times between 2002 and 2009 were analyzed. Incident cancer cases were tracked from 2010 to 2018. Patterns of alcohol drinking, smoking, BMI, and physical activity and latent classes based on trajectories of smoking, alcohol drinking, BMI, and physical activity were identified. Among 2,735,110 adults (1,787,486 men and 947,624 women), 111,218 (69,089 men and 42,129 women) developed incident cancer. Six latent classes of lifestyle behavior were identified, with Class 1 (healthy class) involving only 0.2% of men and 0.5% of women. The highest risk class in males tended to be steady light drinkers and steady moderate smokers, have steady low frequency of physical activity, and be obese. This class showed a 1.47 times higher (95% CI = 1.29–1.69) risk of all cancers than did the healthy class. Among women, there was only an association between the highest risk class (tendency to be non-drinkers, light smokers) and colorectal cancer (HR = 1.70, 95% CI = 1.02–2.83). Only a small percentage of participants maintained a long-term healthy lifestyle. Identifying classes of behavior combinations and their links to cancer development is therefore critical for cancer prevention.

## Introduction

More than half of cancer cases in the past two decades could have been prevented by applying current knowledge. Despite significant advances in the early diagnosis and treatment of cancer, cancer prevention remains one of the most effective strategies for cancer control. Tobacco use, alcohol consumption, inactivity, and obesity are modifiable causes of cancer^1,2^. Epidemiological researches usually pinpoint the association between single lifestyle behaviors and the risk of developing cancer. Although these approaches are crucial for exploring risk factors and the strength of associations in various populations^3–5^, they are unable to determine the combined effect of lifestyle behaviors.

An alternative strategy is to examine the effects of a combination of lifestyle factors on cancer risk. Combinations of lifestyle behaviors may lead to different cancer risks. Given the scarcity of cancer prevention resources, prevention strategies that target populations with similar behaviors may be more cost-effective. For the investigation of lifestyle behavior combinations, an analytic approach that identifies the most common behavioral characteristics is required. Latent class analysis has been used to identify homogenous subgroups of persons within a heterogeneous population. Although some studies have used latent class analysis to determine the association of lifestyle behavior with cancer occurrence, the majority of behaviors are only measured at one time point^6^. However, it is important to recognize that lifestyle behaviors can change over time^7^. Therefore, in this study, we implemented a longitudinal approach by tracking and assessing participants' lifestyle behaviors. This allowed us to capture the dynamic nature of these behaviors and understand how they may influence cancer risk.

Cancer is the main cause of death in the Republic of Korea, accounting for 26.5% of all deaths^8^. Males are most likely to be diagnosed with stomach, colon, lung, prostate, and liver cancer, while females are more likely to be diagnosed with stomach, colorectal, lung, breast, and thyroid cancers^9^. In Korea, smoking causes 22.7% of cancer deaths and 11.8% of new cases of cancer^10^. Although the smoking rate in men in Korea has decreased from 66.3% in 1998 to 36.7% in 2018, it is still relatively higher than in other developed countries^11^. With respect to alcohol consumption, in 2018, 60.6% of Korean adults drank alcohol in the last month, and 50.8% of men and 26.9% of women reported excessive drinking^12^. For physical activity, in 2020, there were 54.4% of adults and 94.1% of adolescents did not engage in sufficient physical activity. The prevalence of sufficient aerobic physical activity has decreased, from 58.3% in 2014 to 45.6% in 2020^13^. In addition, between 2009 and 2018, the prevalence of obesity and abdominal obesity in Korea’s adult population increased steadily^14^.

This study aimed to identify the latent classes based on lifestyle behavior trajectories and to investigate the association between these latent classes and cancer risk. Towards this goal, lifestyle behaviors were measured repeatedly, and a group-based trajectory model was created to identify the main course of lifestyle behaviors. Therefore, this study was a combination of both trajectory analysis and latent class analysis methods.

## Methods

### Study design and data source

This prospective study used data collected by the National Health Insurance Service (NHIS), a single-payer insurance program in Korea, from 2002 to 2018. The NHIS provides nationwide health examination to insured adults biennially. The NHIS general health screening database comprises data from health examinations and results of biochemical tests, as well as demographic information.

### Study population

**Figure 1:**
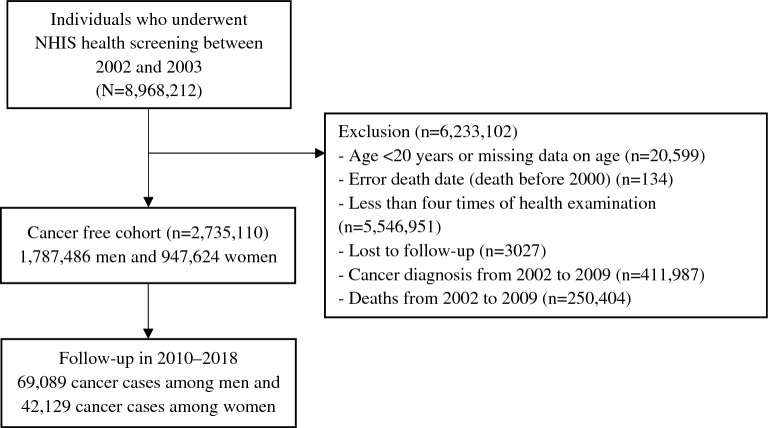
Participant selection flowchart.

We utilized a customized database ran by the NHIS, which covered data of 8,968,212 men and women participating in the 2002–2003 general health examination and followed up through 2018. This study included people who attended four sequential examinations (2002–2003, 2004–2005, 2006–2007, and 2008–2009). Among them, 6,233,102 individuals were excluded owing to age < 20 years, invalid death date, health examination less than four times, cancer diagnosis in 2002–2009, death in 2002–2009, and loss to follow-up. Cancer patients were excluded to form a cancer-free cohort. Finally, 2,735,110 participants (1,787,486 men and 947,624 women) were included in the analysis. Figure 1 describes the study flowchart.

### Measurements

#### Lifestyle behaviors and covariates

Four lifestyle behaviors, namely, alcohol drinking, smoking, body mass index (BMI), and physical activity, were measured four times between 2002 and 2009. Data on alcohol drinking, smoking, and physical activity were collected using a self-administered questionnaire. Smoking was divided into five levels: non-smokers, ex-smokers, 1–9 cigarettes per day, 10–19 cigarettes per day, and ≥ 20 cigarettes per day. Alcohol consumption was categorized into four groups (non-drinkers/1–2 times per week/3–4 times per week/almost every day). The frequency of physical activity was measured using a questionnaire as part of the general health examination, the main question used was: “How many times per week do you perform exercise that makes you sweat?”, and the five responses were none, 1–2 times, 3–4 times, 5–6 times, or almost every day. Due to the low number of individuals in the daily exercise group, we reclassified the physical activity into four categories: none per week, 1–2 times per week, 3–4 times per week, and ≥ 5 times per week. BMI was calculated with directly measured weights and heights and categorized into four groups based on the Guideline for the Management of Obesity of the Korean Society for the Study of Obesity as normal (18.5–22.9 kg/m^2^), overweight (23–24.9 kg/m^2^), obese (25–29.9 kg/m^2^), and severely obese (≥ 30 kg/m^2^)^15^.

Among men, each behavior was categorized into five distinct trajectory groups. Alcohol consumption trajectories included non-drinkers, new drinkers (increased frequency from none to 1–2 times per week), decrease light drinkers (decreased frequency from 1–2 times per week to none), steady light drinkers (maintained frequency of 1–2 times per week), and steady heavy drinkers (maintained frequency of almost every day). For smoking, the trajectories consisted of the following groups: never smokers, new current smokers (transitioning from non-smokers to smoking 1–9 cigarettes per day), decreased light smokers (transitioning from smoking 1–9 cigarettes per day to former smokers), steady moderate smokers (maintaining a smoking habit of 10–19 cigarettes per day), and steady heavy smokers (maintaining a smoking habit of more than 20 cigarettes per day). Regarding physical activity, the trajectory groups were categorized as follows: none per week, beginners (increased frequency from none to 1–2 times per week), decreased low frequency (transitioning from 1–2 times per week to none), steady low frequency (maintaining physical activity 1–2 times per week), and active (maintaining physical activity from 3–4 times to almost every day per week). The BMI trajectory groups consisted of normal weight, increase to overweight, overweight, obesity, and severely obesity. In the case of women, alcohol consumption, BMI and physical activity were also categorized into five distinct trajectory groups and the pattern of these lifestyle behaviors were similar with men. Alcohol consumption trajectories were defined as non-drinkers, new drinkers (increased frequency from none to 1–2 times per week), decreased light drinkers (decreased frequency from 1–2 times per week to none), steady light drinkers (maintained frequency of 1–2 times per week), and steady heavy drinkers (maintained frequency of almost every day). Physical activity trajectories were grouped as follows: none per week, beginners (increased frequency from none to 1–2 times per week), decreased low frequency (transitioned from 1–2 times per week to none), steady low frequency (maintained physical activity 1–2 times per week), and active (maintained physical activity from 3–4 times to almost every day per week). BMI trajectories consisted of normal weight, slightly overweight, overweight, obesity, and severely obesity. As for smoking among women, the trajectories comprised only two groups: never smokers and light smokers (maintaining a smoking habit of 1–9 cigarettes per day).

Covariates were collected at baseline (2002–2003) and included age, income (in quintiles, from the lowest to highest income), and status of chronic viral hepatitis B and C.

### Case identification

We selected the top five cancers among men (gastric, colorectal, lung, prostate, and liver cancers) and top five cancers among women (thyroid, breast, gastric, colorectal, and lung cancers) in Korea in 2017^9^. Cancer incident cases were identified using the International Classification of Disease 10th edition (ICD-10) codes and the registration code (“V193”), which was used to expand the benefit coverage for cancer patients, from 2010 to 2018. The ICD-10 codes for the individual cancer sites were as follows: gastric, C16; colorectal, C18–C20; liver, C22; lung, C33–C34; breast cancer, C50; prostate, C61; and thyroid, C73.

### Statistical analysis

Firstly, group-based trajectory modeling was performed to identify the courses of alcohol consumption, smoking, BMI, and physical activity among the 1,787,486 men and 947,624 women over four times during 2002–2009.

To classify lifestyle behaviors classes, we implemented two steps. In the first step, we selected a class of individuals who exhibited a set of healthy behaviors consistently from 2002 to 2009. Specifically, we identified individuals who did not smoke, did not consume alcohol, did exercise more than 5 times per week, and maintained a normal weight during this period. By establishing this healthy reference class, we created a baseline for comparison and a standard against which the other behavior classes could be evaluated. In the second step, we performed latent class analysis on the remaining participants who did not meet the criteria for the healthy class. Latent class analysis was used to identify latent classes based on the defined trajectories of smoking, alcohol drinking, BMI, and physical activity. Trajectory analysis and latent class analysis were used to identify distinct subgroups within populations that shared particular features. While latent class analysis determined the subgroup at a time, trajectory analysis described the change of a measured variable over time. Instead of attempting to identify every potential profile, both techniques condensed data into the fewest number of classes or trajectories possible. According to posterior probabilities, individuals were assigned to a single class, each of which had a distinctive behavioral profile.

The analysis of the group-based trajectory modeling was performed using the SAS package PROC TRAJ. To choose the best fit model, we followed the tutorial of Andruff et al.^16^. poLCA package in R was used for latent class analysis, and the model fit was evaluated based on Bayesian Information Criterion (BIC), in which a smaller BIC value indicated a better fit. The association between latent classes of lifestyle behaviors and cancer incidence in specific sites were analyzed using the Cox proportional hazard model. Censored cases were defined as those who had no event of interest or died by the end of 2018. Time-to-event (year) was the period from January 1, 2010 to the earliest of the following dates: cancer diagnosis date, censored date, or end of 2018. To adjust for confounding, covariates, consisting of age, income, and the status of chronic viral hepatitis B and C (ICD10: B18) (particularly for liver cancer), were included in the Cox models. All statistical analyses were performed using SAS 9.4 and R software.

### Ethics approval

As this study used anonymous secondary data, it was exempt from review by the Institutional Review Board of the National Cancer Center, Korea (NCC2022-0180). Consent was waived for the same reason.

### Investigation guidelines

This study was designed and conducted according to the Strengthening the Reporting of Observational Studies in Epidemiology (STROBE) reporting guideline.

## Results

The mean age of the male and female participants was 40.5 (SD = 10.6) years and 45.1 (SD = 12.4), respectively. Overall, 48.6% and 36.5% of women and men had normal weight, respectively. The proportion of current smokers at baseline (46.3% vs 1.2%) and alcohol drinkers (43.0% vs 6.9%) were markedly higher in men than in women. Meanwhile, the rate of physical inactivity was lower in men than in women (43.2% vs 66.1%). Table 1 shows the general characteristics of the study population.

**Table 1:** General characteristics of study participants at baseline.

Characteristics	Male		Female	
	n	%	n	%
Age group				
20–29	280,728	15.7	131,871	13.9
30–39	598,249	33.5	149,956	15.9
40–49	573,194	32.1	328,783	34.8
50–59	239,993	13.4	221,163	23.4
≥ 60	93,316	5.2	113,985	12.0
Body mass index				
Normal weight	653,178	36.5	460,468	48.6
Overweight	512,850	28.7	228,740	24.1
Obese	579,261	32.4	233,429	24.6
Severely obese	42,197	2.4	24,987	2.6
Income level				
Lowest quintile	141,460	7.9	187,104	19.7
Second quintile	235,958	13.2	167,275	17.7
Third quintile	462,894	25.9	200,184	21.1
Fourth quintile	456,705	25.6	184,544	19.5
Highest quintile	490,469	27.4	208,517	22.0
Smoking				
Non-smokers	670,393	37.5	925,720	97.7
Ex-smokers	289,863	16.2	10,141	1.1
1–9 cigarettes/day	178,116	10.0	7961	0.8
10–19 cigarettes/day	493,230	27.6	3199	0.3
20–39 cigarettes/day	150,303	8.4	553	0.1
≥ 40 cigarettes/day	5581	0.3	50	0.0
Alcohol consumption				
Non-drinkers	1,018,960	57.0	882,199	93.1
1–2 times per week	557,296	31.2	54,355	5.7
3–4 times per week	160,881	9.0	7032	0.7
Almost everyday	50,349	2.8	4038	0.4
Physical activity				
No	772,525	43.2	626,789	66.1
1–2 times per week	663,444	37.1	178,501	18.8
3–4 times per week	222,065	12.4	74,785	7.9
5–6 times per week	47,713	2.7	20,399	2.2
Almost everyday	81,739	4.6	47,150	5.0
Chronic viral hepatitis B or C				
No	1,586,227	88.7	836,760	88.3
Yes	201,259	11.3	110,864	11.7

**Figure 2:**
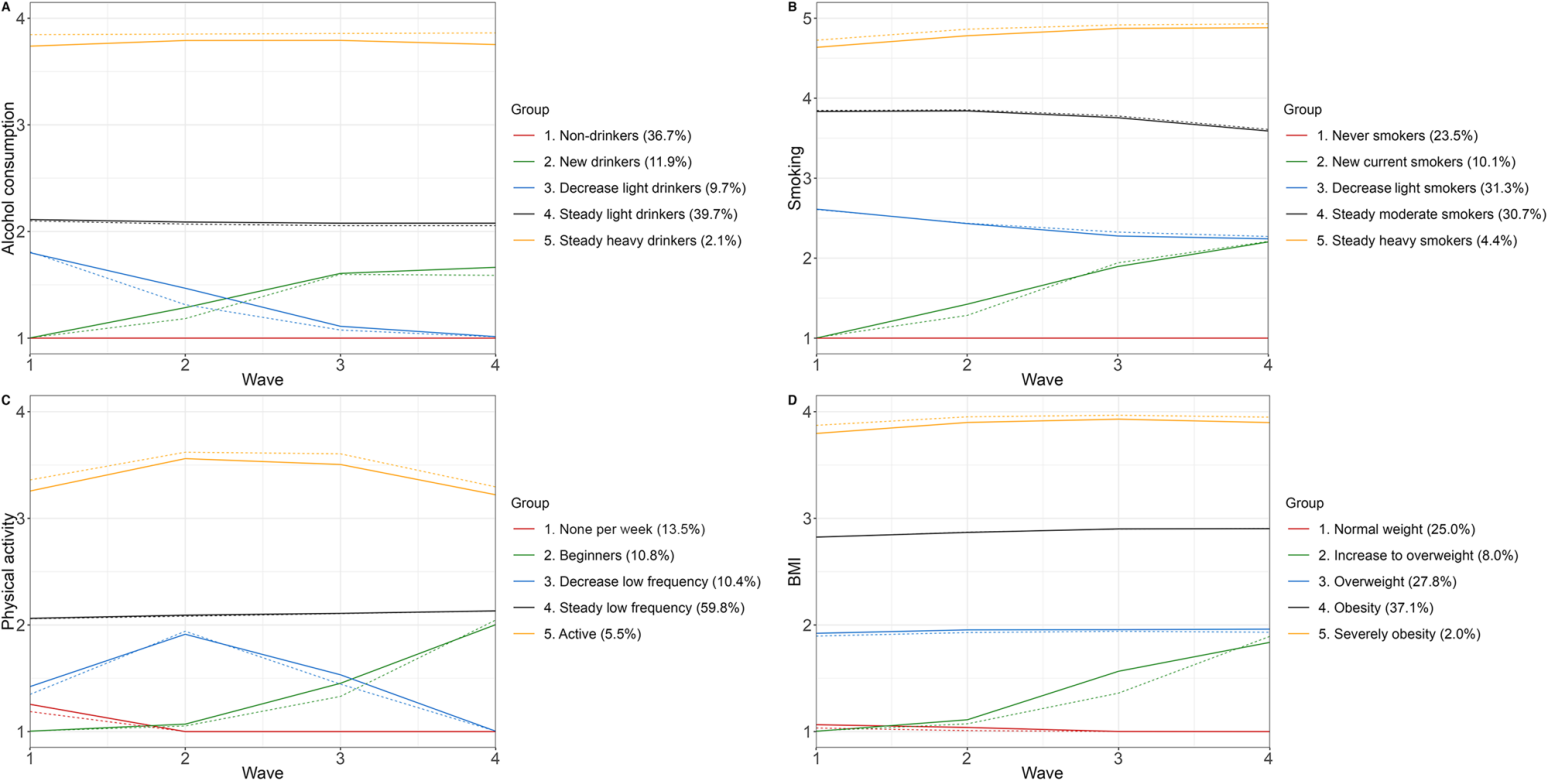
Trajectories of lifestyle behaviors among men.

Figure 2 illustrates the trajectory groups of alcohol consumption, smoking, physical activity, and BMI among men. Each behavior was divided into five distinct trajectory groups. The alcohol consumption trajectory groups consisted of non-drinkers (36.7%), new drinkers (11.9%), decreasing light drinkers (9.7%), steady light drinkers (39.7%), and steady heavy drinkers (2.1%). Similarly, the smoking trajectory groups included never smokers (23.5%), new current smokers (10.1%), decreasing light smokers (31.3%), steady moderate smokers (30.7%), and steady heavy smokers (4.4%). Additionally, the physical activity trajectory groups encompassed inactive individuals (13.5%), beginners (10.8%), decreasing low frequency participants (10.4%), steady low frequency participants (59.8%), and active individuals (5.5%). Lastly, the BMI trajectory groups comprised normal weight individuals (25.0%), individuals increasing to overweight (5.9%), overweight individuals (24.9%), individuals with obesity (14.6%), and individuals with severe obesity (28.3%).

**Figure 3:**
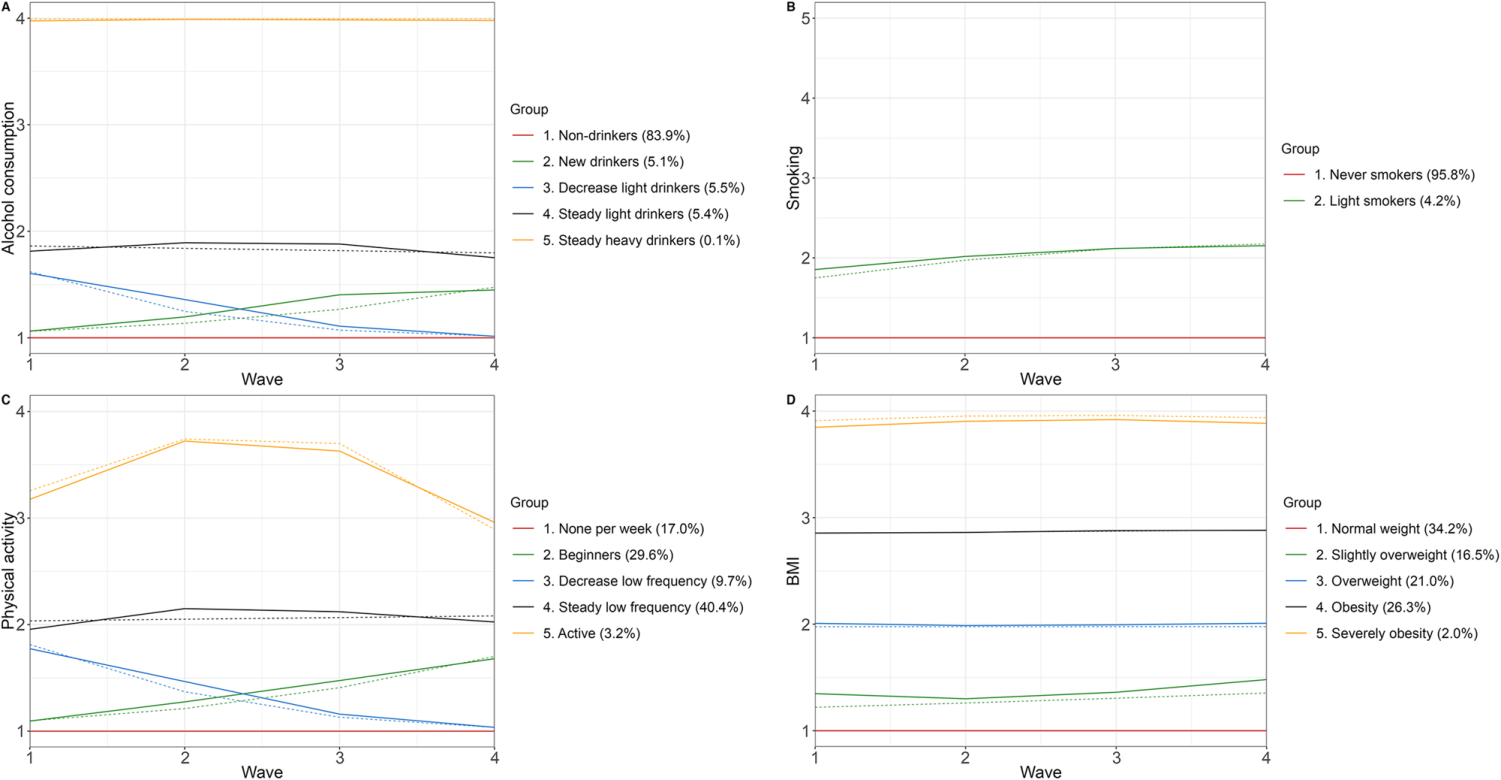
Trajectories of lifestyle behaviors among women.

Figure 3 displays the trajectory groups of alcohol consumption, smoking, physical activity, and BMI among women. We identified five trajectory groups for alcohol consumption, physical activity, and BMI, and two trajectory groups for smoking. The alcohol consumption trajectory groups consisted of non-drinkers (83.9%), new drinkers (5.1%), decreasing light drinkers (5.5%), steady light drinkers (5.4%), and steady heavy drinkers (0.1% ). The smoking trajectory groups included never smokers (95.8%), and light smokers (4.2%). Additionally, the physical activity trajectory groups encompassed inactive individuals (17.0%), beginners (29.6%), decreasing low frequency participants (9.7%), steady low frequency participants (40.4% of participants), and active individuals (3.2%). Lastly, the BMI trajectory groups comprised normal weight individuals (34.2%), slightly overweight (16.5% of participants), overweight individuals (21.0%), individuals with obesity (26.3%), and individuals with severe obesity (2.0%).

**Figure 4:**
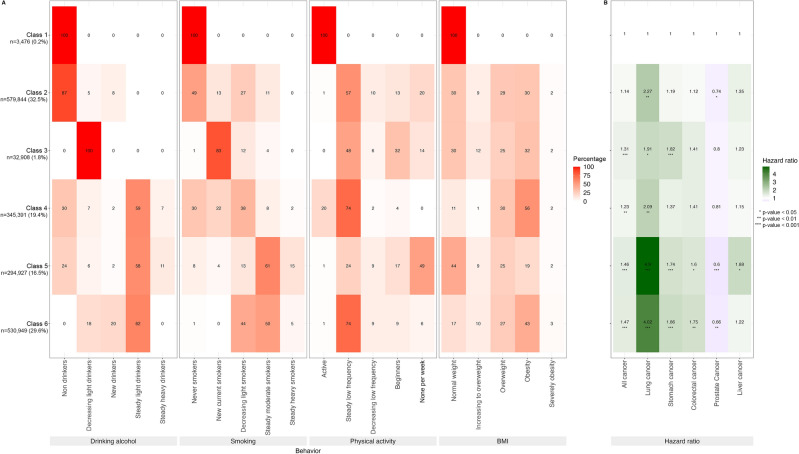
Latent classes of alcohol consumption, smoking, physical activity, and BMI trajectories and the association of these classes with cancer incidence among men. (**A**) Percentage men in each trajectory category for each behavior in each class. Latent classes are represented by rows, and each behavior trajectory is represented by a column. For each behavior, the left column represents the lower risk trajectory, and the right column represents the higher risk trajectory. (**B**) Hazard ratios for each of the five types of cancer and for overall cancers for each class with the healthy class (class 1) as reference and adjusted for age, income, and chronic viral hepatitis B or C for liver cancer.

Figure 4 shows the latent classes of alcohol consumption, smoking, physical activity, and BMI trajectories and the association of these classes with cancer incidence among men. There were six classes of lifestyle trajectories as follows: Class 1 (healthy group, non-drinkers, never smokers, active physical activity, normal weight) included 0.2% of the participants; Class 2 (tend to be non-drinkers, never and light smokers, steady low frequency of physical activity), 32.5%; Class 3 (tend to decreasing light drinkers, new current smokers, steady low frequency of physical activity), 1.8%; Class 4 (tend to be steady light drinkers, decreasing light smokers, steady low frequency of physical activity, and obese), 19.4%; Class 5 (tend to be steady light drinkers, steady moderate smokers, steady low frequency of physical activity, and obese), 29.6%; and Class 6 (tend to be steady light drinkers, steady moderate smokers, inactive physical activity, and normal weight), 16.5%.

With class 1 as reference, the risk of cancer was 1.46 (95% CI, 1.28–1.68) for class 6; 1.47 (95% CI, 1.29–1.69) for class 5; 1.23 (95% CI, 1.08–1.41) for class 4; and 1.31 (95% CI, 1.12–1.52) for class 3. Among the top 5 cancers in men, the risk of developing lung cancer was highest. The risks of lung cancer in Classes 5 and 6, with class 1 as reference, were 4.02 (95% CI, 2.33–6.95) and 4.90 (95% CI, 2.84–8.46), respectively. Meanwhile, Classes 2, 3, 5, and 6 showed a negative association with prostate cancer.

**Figure 5:**
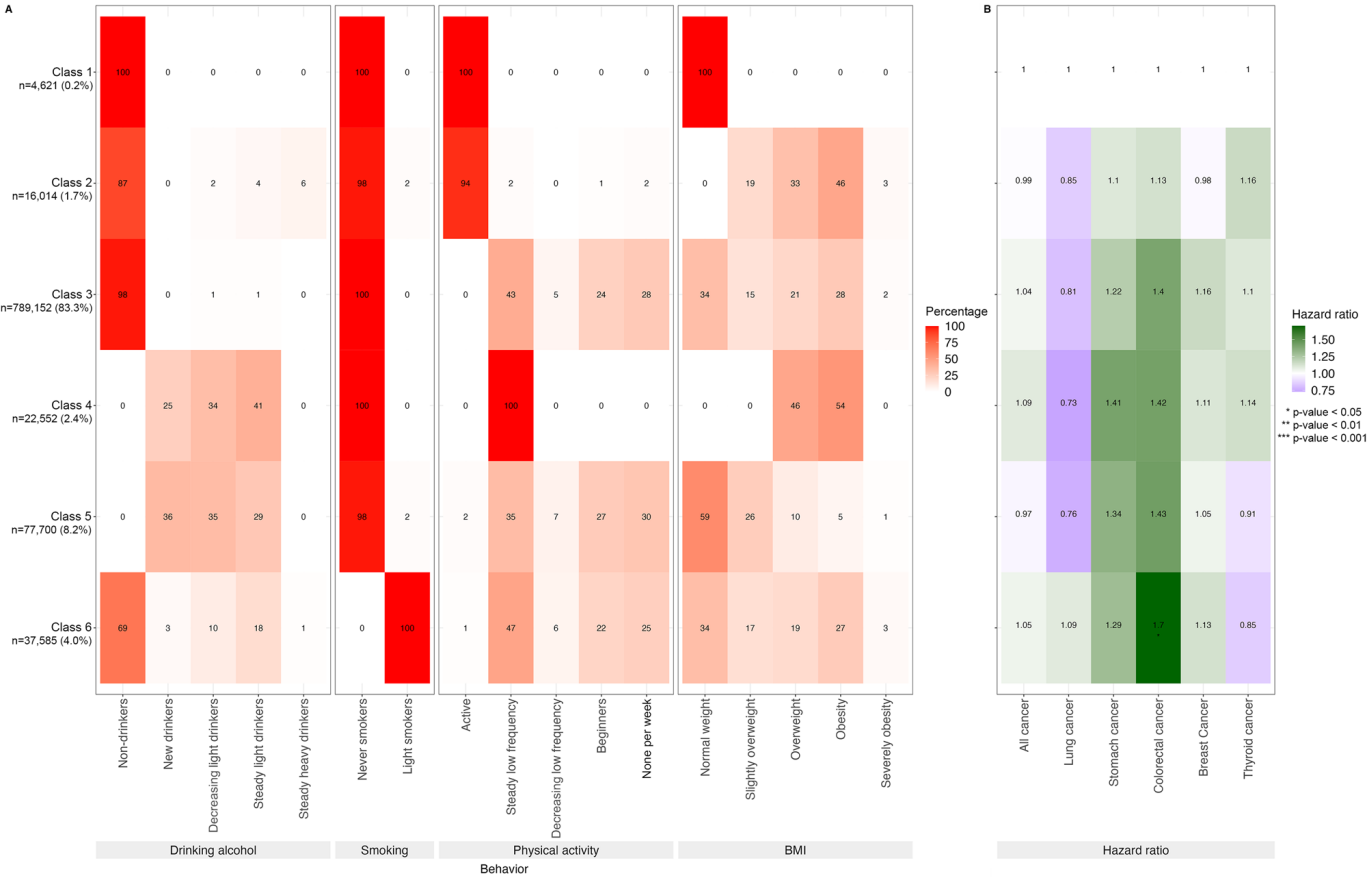
Latent classes of alcohol consumption, smoking, physical activity, and BMI trajectories and the association of these classes with cancer incidence among women. (**A**) Percentage of women in each trajectory category for each behavior in each class. Latent classes are represented by rows, and each behavior trajectory is represented by a column. For each behavior, the left column represents the lower risk trajectory, and the right column represents the higher risk trajectory. (**B**) Hazard ratios for each of the five types of cancer and for overall cancers for each class, with the healthy class (class 1) as reference, adjusted for age, income, and chronic viral hepatitis B or C for liver cancer.

Figure 5 shows the latent classes of alcohol consumption, smoking, physical activity, and BMI trajectories and the association of these classes with cancer incidence among women. A total of six classes of lifestyle trajectories were identified as follows: Class 1 (healthy group, non-drinkers, never smokers, active physical activity, normal weight) included 0.5% of the population; Class 2 (tend to be non-drinkers, never smokers, active physical activity, and obesity), 1.7%; Class 3 (tend to non-drinkers, never smokers, steady low frequency of physical activity), 83.3%; Class 4 (tend to be steady light drinkers, never smokers, steady low frequency of physical activity, and overweight or obese), 2.4%; Class 5 (tend to be new drinkers or light drinkers, never smokers, and normal weight), 8.2%; and Class 6 (tend to be non-drinkers, light smokers), 4.0%.

With class 1 as references, there was no significant association between other latent classes and overall incidence of cancers. Among the top 5 cancers in women, only class 6 showed an association with colorectal cancer. The risk of developing colorectal cancer in class 6 was 1.7 (95% CI, 6.94–8.14) (Supplementary Tables).

## Discussion

We conducted this study to identify the combinations of lifestyle behaviors and determine the associations of these combinations with the risk of all cancers combined and the top five cancers in both men and women in Korea. The combination of trajectory analysis and latent class analysis in this prospective study identified six classes of lifestyle behaviors measured four times from 2002 to 2009 in both sexes. To more easily compare the risk of developing cancer among these classes, we defined Class 1 as healthy people who did not smoke, did not drink alcohol, were physically active, and were of normal weight from 2002 to 2009. There were only 0.2% of men and 0.5% of women who belonged to this class. This demonstrated that only a small proportion of the population can maintain a completely healthy lifestyle for an extended period (8 years), and most study participants engaged in at least one unhealthy behavior.

For men, in the remaining five classes, no group included all high-risk behaviors. The classes tended to exhibit a mix of healthy and unhealthy behaviors, thereby reducing combined risk. With Class 1 as reference, most classes, except for Class 2, had a significant association with cancer risk. Out of the remaining five classes, Class 2 displayed relatively healthier lifestyle behaviors. A significant 32.5% of the participants belonged to this particular class, indicating a higher proportion compared to the other classes. Class 2 comprises individuals with mixed lifestyle patterns. While a majority are non-drinkers and never smokers, a small percentage has started their alcohol and tobacco use. In terms of physical activity, a diverse range is observed, with some individuals being inactive, while others show varying levels of frequency and engagement. Weight distribution also varies within this class, with a notable proportion falling into normal weight, overweight, and obesity. The cancer risk in this class did not show a significant difference from that in Class 1. Meanwhile, Classes 5 and 6 were the two most at-risk classes. Despite the opposing exercise behavior and weight in these two classes, they shared common features of higher alcohol and smoking consumption than did the other classes and maintained this unhealthy habit for a long time. The results from Appendix Table 1 indicate that individuals who maintained alcohol and tobacco use over an extended period faced a greater risk of developing cancer. Class 5 was characterized by individuals who maintained a consistent pattern of light alcohol consumption. A significant portion of individuals were categorized as steady light drinkers, while a small percentage were steady heavy drinkers. Smoking behavior in Class 5 revealed that most individuals were steady moderate smokers, with a minority being steady heavy smokers. Physical activity levels predominantly leaned towards inactivity, with a substantial number of individuals not participating in any form of physical activity. In terms of weight distribution, a significant proportion had a normal weight, while others were transitioning to overweight or fell into the overweight category. Moreover, a notable percentage of individuals in this class were classified as obese or severely obese. Class 6 primarily consisted of individuals who maintained a consistent pattern of light alcohol consumption, with no individuals identified as steady heavy drinkers. Regarding smoking behavior, a considerable number of individuals were steady moderate smokers, while a minority were steady heavy smokers. Physical activity levels exhibited variability, ranging from regular physical activity to a steady low frequency, reducing frequency, beginner level, or inactivity. Weight distribution in this class showed variations, with individuals falling into different weight categories, including normal weight, transitioning to overweight, overweight, obesity, and severe obesity. The risk of overall cancers and the top 5 cancers (except for prostate cancer) were the highest in these two classes. Particularly, the hazard ratios for lung cancer were 4.90 and 4.02 in Classes 5 and 6, respectively. Previous studies have also shown that long-term and heavy alcohol intake, as well as smoking, increased the risk of cancer. A meta-analysis of 254 studies also found a strong association between smoking and cancer risk, particularly among moderate and heavy smokers^17^. Similarly, heavy drinkers have a greater risk of some cancers than have non-drinkers and occasional drinkers, and the dose-risk association is evident^18^. In addition, the combination of alcohol drinking and smoking has also been shown to increase the risk of cancer^19–21^. Meanwhile, Class 3 predominantly comprised individuals who demonstrated a declining trend in their light alcohol consumption. Smoking behavior in this class indicated that a majority were new current smokers, while a portion showed a reduction in their light smoking habits. Physical activity levels displayed variability, with individuals engaging in a consistent low frequency of physical activity, being beginners in terms of physical activity, or being inactive. Regarding weight distribution, individuals in Class 3 included those with a normal weight, transitioning to overweight, overweight, obesity, or severe obesity. On the other hand, Class 4 consisted of individuals with distinct characteristics. Some individuals in this class were non-drinkers, while a significant proportion adhered to a steady pattern of light drinking. Regarding smoking behavior, individuals in Class 4 included never smokers, new current smokers, and those reducing their light smoking habits. Physical activity levels ranged from regular physical activity to a steady low frequency. Weight distribution varied, with individuals falling into different weight categories, such as normal weight, overweight, and obesity. While the overall cancer risk in Class 3 and Class 4 was lower than that in Classes 5 and 6, it remained higher than that in Class 1. This result was consistent with that of a cohort study in Korea in which those who increased their alcohol use were found to have a greater risk of alcohol-related malignancies and all cancers than did those who drank at a steady level^22^. Increasing the number of cigarettes smoked has also been shown to increase the risk of cancer^17,23^.

Notably, the five classes with unhealthy habits in this study had a higher risk of lung cancer than did the healthy class. Smoking is the leading cause of lung cancer. Although the prevalence of moderate or heavy smokers was low or negligible in Classes 2, 3, and 4, these classes nevertheless included new current smokers or light smokers. Compared with never smokers, those who were new current smokers or had reduced or quit smoking were still at higher risk. Similar results were found by a study about smoking trajectory and cancer risk in Korea^24^. In contrast to the other types of cancer, prostate cancer had a negative association with all classes. Several studies have investigated the causes and risk factors for developing prostate cancer. However, the findings on the association between lifestyle behaviors and prostate cancer risk have been conflicting, with no clear link found to prostate cancer^25^. A competing risk of death can be a reason of the inverse association. People with unhealthy behaviors may have a higher chance of premature death, die from other issues before developing prostate cancer, and may have a lower chance to develop prostate cancer.

For instance, before prostate cancer diagnosis, smokers in this study were more likely to die earlier from smoking-related illnesses such as coronary heart disease, chronic obstructive pulmonary disease, and smoking-related cancers. Participants with a past history of other cancers, including cancers related to alcohol use and smoking, were excluded from the analysis of prostate cancer risk. In addition, the participants’ behaviors may have changed before the study period. People who are diagnosed with health problems tend to change their unhealthy behaviors, and this could happen before the data collection period for this study. The findings were consistent with those of a 2018 study in Korea that used a similar dataset from the National Health Insurance Corporation in Korea. Compared to heavy drinkers and active smokers, patients who did not consume alcohol or smoke were estimated to have a higher prostate cancer risk^26^. The authors proposed several hypotheses to explain their findings, which were relatively consistent with our interpretation.

For women, except for those in Class 1 (healthy class), those in the remaining classes had relatively different characteristics from men. In particular, Class 3 consisted of 83.3% female participants. This class primarily comprised individuals who abstained from alcohol consumption. Moreover, all individuals in Class 3 had never smoked. In terms of physical activity, none of the individuals engaged in active physical activity, while a significant portion maintained a consistent but low frequency of physical activity. Additionally, there were individuals who gradually reduced their frequency of physical activity, some who were just beginning to engage in physical activity, and a notable portion who remained inactive. Regarding weight distribution, a considerable number of individuals in Class 3 had a normal weight. However, there were individuals who were transitioning towards overweight, as well as others who fell into the overweight category. Furthermore, a significant proportion of individuals in this class were classified as obese. Women in Class 4 and 5 were also never smokers but tended to consume more alcohol than did the women in the other groups; meanwhile, class 6 involved female smokers. Most of these latent classes did not show a statistically significant difference in both risks of combined cancers and specific cancers. The limited number of cancer cases observed in each class may have contributed to these results. Only class 6 showed a significant association with colorectal cancer. The most apparent difference between Class 6 and the other classes was that all the women in this class were former or current smokers. According to Appendix Table 2, light female smokers had a 1.12 times higher risk of colorectal cancer compared to the group of female non-smokers. Smoking has been shown to be a risk factor for colorectal cancer in both sexes^27,28^. One notable finding of this study is that there appeared to be a lower risk of lung cancer in classes other than Class 1 among women, although these results did not reach statistical significance. This can be explained by the smoking rate in women in our study was relative low (1.2%) and may have less impact on lung cancer incidence. Other factors, such as environmental exposures (including secondhand smoking, radon exposure, and cooking oil fumes)^29^, which were not accounted for in this study, could potentially have a more significant influence on the risk of developing lung cancer.

The strength of this study is that we have identified the main behavioral groups of the population and assessed the risk of cancer in these groups. The findings can be useful for selecting priority groups for cancer prevention. In addition, unlike most previous studies that measured behavioral combinations at one time point^30^, the current study determined the trajectories of behaviors measured four times from 2002 to 2009 and used latent class analysis to aggregate the results from these trajectories. This improved the objectivity of the evaluation of behaviors. This study examined the association between changes in multiple behaviors over time and the risk of developing cancer. The findings indicated that prolonged engagement in risk behaviors was associated with the highest cancer risk. Conversely, there was potential for risk reduction when individuals made improvements or changes towards healthier behaviors, such as decreasing alcohol consumption, decreasing smoking intensity, and initiating physical activity. For instance, the risk of lung cancer was found to be more than four times higher in Class 5 and Class 6 compared to Class 1, which had a higher proportion of individuals maintaining risk behaviors. However, the HR decreased to approximately 2 in Class 2 and Class 3, which had an increased proportion of individuals who had successfully reduced their alcohol consumption, initiated smoking, and engaged in physical activity. These findings suggested that modifying behaviors towards healthier choices could potentially mitigate the risk of cancer. Therefore, interventions targeting behavior changes and promoting healthier lifestyles may play a crucial role in reducing cancer incidence and improving public health outcomes.

However, this study had a number of limitations. First, only people who had four consecutive health examinations were included. The characteristics of the study population may be different from those of the general population, and thus, the results should be interpreted with caution. Second, behavior status may have been misclassified owing to the nature of self-reported data. Especially for women, smoking and alcohol consumption tend to be underreported given the socially conservative culture. A study showed that cotinine-verified smoking rate is double of the self-reported smoking rate for women^31^. The prevalence of current smoking (1.2%) and alcohol consumption (6.9%) among women in the current study was lower than that in a 2005 national survey (smoking: 5.7%; alcohol consumption 37.0%)^11^. The differences in prevalence may due to the different populations, and underreporting cannot be ruled out. Third, because lifestyle behaviors were only examined during this study period, the findings may not be conclusive about the long-term impact of changes in lifestyle behaviors on cancer risk. Fourth, alcohol consumption was categorized only based on frequency, not amount of alcohol consumed. However, frequency may be a more reliable factor than is the amount of alcohol among the Korean population^32^. Fifth, the Class 1, a reference group, has a relatively small sample size (i.e. 0.2% in males and 0.5% in females) which may occur statistical issues such as unstable estimates, increased risk bias, and inadequate adjustment for covariate. However, the number of participants in the Class 1 group were 3467 men and 4621 women, not too small to yield reliable estimates. Physical activity was also categorized based on frequency without consideration of intensity. Lastly, we cannot account for all potential confounding factors when assessing cancer risk. Diet, family history, genetic factors, occupational exposures, and other health conditions were not included in our analysis.

## Conclusion

A large proportion of the study population have one or more behavioral risk factors for cancer, and only a small proportion of individuals maintained healthy behaviors for at least 8 years. In general, women have a healthier lifestyle than men. No class exhibited all unhealthy behaviors, and most classes were combinations of some unhealthy and healthy behaviors, thereby reducing their overall cancer risk. In men, those in classes that tended to drink alcohol frequently and smoke for a long time have the highest risk of all cancers and cancers in the lung, stomach, colorectum, and liver. The colorectal cancer risk is elevated in women who tended to be non-drinkers and light smokers. Therefore, cancer prevention entails the identification of classes of behavior combinations and their links to cancer development.

### Supplementary Information

Supplementary Tables.

## Data Availability

The datasets generated and analyzed during the current study cannot be shared because NHIS prohibits the transfer, rental, or sale of the database to third parties except for researchers who have been approved for access. NHIS data are available upon request from the National Health Insurance Sharing Service, https://nhiss.nhis.or.kr/.
