# Supplementary material for: Combinations of lifestyle behaviors and cancer risk among Korean adults
Source: Sci Rep. 2023 Aug 23;13:13765. doi: 10.1038/s41598-023-40819-w (PMC10447503; doi:10.1038/s41598-023-40819-w)
Supplement: Supplementary file 1 — Supplementary Tables. [file 41598_2023_40819_MOESM1_ESM.docx]

Appendix Table 1. Lifestyle behaviors trajectories and all cancer and top five cancers incidence among men

| Cancer site | Adjusted hazard ratio (95% CI) | | | | | |
| --- | --- | --- | --- | --- | --- | --- |
|  | All cancer | Lung cancer | Stomach cancer | Colorectal cancer | Prostate cancer | Liver cancer |
| Drinking alcohol |  |  |  |  |  |  |
| Non drinkers | ref | ref | ref | ref | ref | ref |
| Decreasing light drinkers | 0.99 (0.96 - 1.01) | 0.92 (0.84 - 1.00) | 1.12 (1.06 - 1.19) | 1.09 (1.02 - 1.17) | 0.94 (0.87 - 1.01) | 1.00 (0.92 - 1.08) |
| New drinkers | 1.06 (1.03 - 1.08) | 0.95 (0.87 - 1.04) | 1.21 (1.14 - 1.28) | 1.15 (1.07 - 1.23) | 0.97 (0.90 - 1.05) | 1.24 (1.15 - 1.34) |
| Steady light drinkers | 1.05 (1.04 - 1.07) | 0.98 (0.94 - 1.03) | 1.17 (1.13 - 1.21) | 1.28 (1.23 - 1.33) | 0.95 (0.92 - 0.99) | 1.02 (0.97 - 1.07) |
| Steady heavy drinkers | 1.18 (1.15 - 1.22) | 1.09 (1.01 - 1.17) | 1.19 (1.11 - 1.27) | 1.36 (1.26 - 1.47) | 0.88 (0.81 - 0.96) | 1.30 (1.18 - 1.43) |
| Smoking |  |  |  |  |  |  |
| Never smokers | ref | ref | ref | ref | ref | ref |
| New current smokers | 1.10 (1.07 - 1.12) | 1.38 (1.29 - 1.48) | 1.21 (1.15 - 1.27) | 1.12 (1.06 - 1.18) | 0.95 (0.90 - 1.00) | 1.19 (1.11 - 1.27) |
| Decreasing light smokers | 1.16 (1.15 - 1.18) | 1.90 (1.80 - 2.01) | 1.33 (1.28 - 1.38) | 1.21 (1.16 - 1.27) | 0.91 (0.88 - 0.95) | 1.22 (1.15 - 1.29) |
| Steady moderate smokers | 1.35 (1.33 - 1.37) | 3.57 (3.37 - 3.78) | 1.63 (1.56 - 1.70) | 1.31 (1.25 - 1.38) | 0.75 (0.71 - 0.79) | 1.52 (1.44 - 1.61) |
| Steady heavy smokers | 1.40 (1.35 - 1.45) | 5.38 (4.92 - 5.87) | 1.72 (1.60 - 1.86) | 1.32 (1.20 - 1.44) | 0.76 (0.67 - 0.86) | 1.15 (1.02 - 1.31) |
| Physical activity |  |  |  |  |  |  |
| Active | ref | ref | ref | ref | ref | ref |
| Steady low frequency | 1.01 (0.99 - 1.03) | 1.08 (1.00 - 1.17) | 0.99 (0.94 - 1.05) | 0.99 (0.92 - 1.06) | 1.00 (0.94 - 1.07) | 1.09 (1.00 - 1.19) |
| Decreasing low frequency | 1.02 (0.99 - 1.06) | 1.12 (1.01 - 1.23) | 0.99 (0.92 - 1.07) | 1.01 (0.92 - 1.10) | 0.95 (0.87 - 1.03) | 1.07 (0.96 - 1.19) |
| Beginners | 1.02 (0.99 - 1.05) | 1.17 (1.07 - 1.28) | 0.98 (0.92 - 1.05) | 1.03 (0.95 - 1.11) | 0.93 (0.86 - 1.01) | 1.07 (0.96 - 1.18) |
| Inactive | 1.00 (0.97 - 1.02) | 1.13 (1.04 - 1.23) | 0.96 (0.90 - 1.03) | 0.98 (0.91 - 1.05) | 0.88 (0.82 - 0.95) | 1.08 (0.98 - 1.19) |
| BMI |  |  |  |  |  |  |
| Normal weight | ref | ref | ref | ref | ref | ref |
| Increasing to overweight | 0.99 (0.97 - 1.02) | 0.90 (0.83 - 0.97) | 0.97 (0.92 - 1.03) | 0.99 (0.93 - 1.07) | 1.02 (0.94 - 1.10) | 0.94 (0.86 - 1.03) |
| Overweight | 1.03 (1.01 - 1.05) | 0.89 (0.85 - 0.93) | 0.99 (0.96 - 1.03) | 1.11 (1.06 - 1.16) | 1.11 (1.06 - 1.16) | 1.01 (0.96 - 1.07) |
| Obesity | 1.10 (1.08 - 1.12) | 0.83 (0.80 - 0.87) | 1.08 (1.04 - 1.12) | 1.22 (1.17 - 1.27) | 1.14 (1.09 - 1.19) | 1.16 (1.10 - 1.22) |
| Severely obesity | 1.30 (1.25 - 1.36) | 0.73 (0.62 - 0.87) | 1.16 (1.04 - 1.30) | 1.47 (1.30 - 1.65) | 1.07 (0.92 - 1.24) | 1.70 (1.49 - 1.94) |

*Note: Hazard ratios were adjusted for age, income, and chronic viral hepatitis B or C for liver cancer*

Appendix Table 2. Lifestyle behaviors trajectories and all cancer and top five cancers incidence among women

|  | Adjusted hazard ratio (95% CI) | | | | | |
| --- | --- | --- | --- | --- | --- | --- |
|  | All cancer | Lung cancer | Stomach cancer | Colorectal cancer | Breast cancer | Thyroid cancer |
| Drinking alcohol |  |  |  |  |  |  |
| Non drinkers | ref | ref | ref | ref | ref | ref |
| Decreasing light drinkers | 0.95 (0.91 - 1.00) | 1.01 (0.82 - 1.25) | 1.23 (1.05 - 1.45) | 1.00 (0.84 - 1.20) | 0.88 (0.80 - 0.97) | 0.88 (0.81 - 0.95) |
| New drinkers | 0.95 (0.91 - 0.99) | 0.87 (0.71 - 1.06) | 0.89 (0.75 - 1.05) | 0.97 (0.83 - 1.13) | 0.89 (0.81 - 0.98) | 0.91 (0.85 - 0.98) |
| Steady light drinkers | 0.98 (0.94 - 1.01) | 1.04 (0.90 - 1.21) | 1.03 (0.91 - 1.18) | 1.07 (0.94 - 1.21) | 0.93 (0.85 - 1.01) | 0.84 (0.78 - 0.91) |
| Steady heavy drinkers | 1.04 (0.88 - 1.22) | 1.22 (0.77 - 1.94) | 1.06 (0.67 - 1.68) | 1.25 (0.84 - 1.86) | 0.89 (0.51 - 1.57) | 0.79 (0.48 - 1.31) |
| Smoking |  |  |  |  |  |  |
| Never smokers | ref | ref | ref | ref | ref | ref |
| Light smokers | 1.01 (0.98 - 1.05) | 1.46 (1.29 - 1.66) | 1.07 (0.94 - 1.22) | 1.12 (1.00 - 1.27) | 0.95 (0.86 - 1.04) | 0.79 (0.72 - 0.86) |
| Physical activity |  |  |  |  |  |  |
| Active | ref | ref | ref | ref | ref | ref |
| Steady low frequency | 1.02 (0.98 - 1.08) | 0.94 (0.80 - 1.12) | 1.05 (0.90 - 1.23) | 1.01 (0.88 - 1.17) | 1.14 (1.00 - 1.30) | 1.00 (0.90 - 1.11) |
| Decreasing low frequency | 0.98 (0.93 - 1.04) | 0.83 (0.67 - 1.03) | 1.06 (0.87 - 1.28) | 0.94 (0.78 - 1.12) | 1.00 (0.85 - 1.17) | 0.94 (0.83 - 1.07) |
| Beginners | 1.03 (0.98 - 1.08) | 0.99 (0.83 - 1.18) | 1.10 (0.93 - 1.29) | 0.96 (0.83 - 1.12) | 1.08 (0.94 - 1.24) | 0.97 (0.87 - 1.08) |
| Inactive | 1.01 (0.96 - 1.06) | 0.95 (0.80 - 1.13) | 1.13 (0.96 - 1.33) | 1.01 (0.87 - 1.17) | 1.02 (0.89 - 1.17) | 0.91 (0.81 - 1.01) |
| BMI |  |  |  |  |  |  |
| Normal weight | ref | ref | ref | ref | ref | ref |
| Slightly overweight | 1.02 (0.99 - 1.04) | 1.02 (0.93 - 1.12) | 1.08 (0.99 - 1.17) | 0.98 (0.90 - 1.06) | 0.91 (0.86 - 0.97) | 1.11 (1.07 - 1.17) |
| Overweight | 1.06 (1.04 - 1.08) | 0.93 (0.85 - 1.01) | 1.10 (1.02 - 1.19) | 1.06 (0.99 - 1.14) | 1.00 (0.95 - 1.05) | 1.22 (1.17 - 1.27) |
| Obesity | 1.10 (1.08 - 1.12) | 0.90 (0.83 - 0.97) | 1.13 (1.05 - 1.21) | 1.10 (1.03 - 1.18) | 1.05 (1.00 - 1.10) | 1.28 (1.23 - 1.34) |
| Severely obesity | 1.20 (1.14 - 1.26) | 0.89 (0.73 - 1.09) | 1.22 (1.03 - 1.43) | 1.24 (1.07 - 1.44) | 1.12 (0.98 - 1.28) | 1.21 (1.08 - 1.35) |

*Note: Hazard ratios were adjusted for age, income*
